# Deep Learning-Based Microscopic Damage Assessment of Fiber-Reinforced Polymer Composites

**DOI:** 10.3390/ma17215265

**Published:** 2024-10-29

**Authors:** Muhammad Muzammil Azad, Atta ur Rehman Shah, M. N. Prabhakar, Heung Soo Kim

**Affiliations:** 1 Department of Mechanical, Robotics and Energy Engineering, Dongguk University-Seoul, 30 Pildong-ro 1-gil, Jung-gu, Seoul 04620, Republic of Korea; muzammilazad@dgu.ac.kr; 2 Department of Mechanical Engineering, COMSATS University Islamabad, Wah Campus, Wah Cantt 47040, Pakistan; atta85@cuiwah.edu.pk; 3 Department of Mechanical Engineering, Changwon National University, 20 Changwondaehak-ro, Uichang-gu, Changwon 51140, Republic of Korea; dr_prabhakar@changwon.ac.kr

**Keywords:** deep learning, microscopic damage, scanning electron microscopy, transfer learning, damage assessment, fiber-reinforced polymers

## Abstract

Fiber-reinforced polymers (FRPs) are increasingly being used as substitutes for traditional metallic materials across various industries due to their exceptional strength-to-weight ratio. However, their orthotropic properties make them prone to multiple forms of damage, posing significant challenges in their design and application. During the design process, FRPs are subjected to various loading conditions to study their microscopic damage behavior, typically assessed through scanning electron microscopy (SEM). While SEM provides detailed insights into fracture surfaces, the manual analysis of these images is labor-intensive, time-consuming, and subject to variability based on the observer’s expertise. To address these limitations, this research proposes a deep learning-based approach for the autonomous microscopic damage assessment of FRPs. Several computationally efficient pre-trained deep learning models, such as DenseNet121, NasNet Mobile, EfficientNet, and MobileNet, were evaluated for their performance in identifying different damage modes autonomously, thus reducing the need for manual interpretation. SEM images of FRPs with five distinct failure modes were used to validate the proposed method. These failure modes include three fiber-based failures such as fiber breakage, fiber pullout, and mixed-mode failure, and two matrix-based failures such as matrix brittle failure and matrix ductile failure. The entire dataset is divided into train, validation, and test sets. Deep learning models were established by training on train and validation sets for five failure modes, while the test set was used as the unseen data to validate the models. The models were assessed using various evaluation metrics on an unseen test dataset. Results indicate that the EfficientNet model achieved the highest accuracy of 97.75% in classifying the failure modes. The findings demonstrate the effectiveness of employing deep learning techniques for microscopic damage assessment, offering a more efficient, consistent, and scalable solution compared to traditional manual analysis.

## 1. Introduction

Fiber-reinforced polymer (FRP) composites offer significantly higher specific strength and stiffness compared to conventional metallic materials, resulting in improved mechanical properties and considerable weight savings [1]. These advantages have led to widespread adoption of FRPs across various industries, including aerospace, automotive, and marine engineering [2]. The specific applications of FRPs in these industries include aircraft components, such as fuselage sections, composite hydraulic cylinders and wing structures, automotive body panels and chassis parts, and marine hulls and decks, where the lightweight and high-strength properties of FRPs enhance performance and fuel efficiency [2,3,4]. However, due to their orthotropic nature, FRPs are susceptible to a range of microscopic damage mechanisms. The microscopic damages refer to a wide range of small-scale defects or damages that occur at the microstructural level within the FRPs [5]. These types of damages are not visible to the naked eye and require sophisticated techniques to be observed and analyzed. Some of the microscopic damages that occur in FRPs are fiber breakage, matrix cracking, debonding, and delamination [6]. Among these, delamination is particularly critical as it can significantly compromise the structural integrity and performance of FRPs [7]. Therefore, significant research has been conducted on detailed analysis of delamination detection in FRPs using physics-based approaches [8,9,10,11] and data-driven approaches [12,13,14]. However, in practical applications, delamination is the consequence of the preliminary damage to the fibers or matrix. Therefore, during material design, it is crucial to not only focus on delamination detection but also to comprehensively assess the initiation and progression of various microscopic damage mechanisms such as fiber breakage and matrix cracking. Such approaches can lead to more effective material design strategies, ultimately enhancing the reliability and performance of FRP structures in demanding environments. Moreover, the damage mechanisms in FRPs are also influenced by the type of fibers, resins, and manufacturing processes, such as pultrusion and vacuum infusion molding, which produce distinct failure modes [15]. Thermosetting and thermoplastic resins also behave differently due to variations in viscosity and interfacial bonding [16]. Additionally, environmental factors like temperature and moisture, combined with mechanical loads, lead to varying damage mechanisms in fiber-reinforced composites [17].

The microscopic damage in FRPs can be assessed through numerous techniques such as micro-computed tomography (μCT), guided waves, acoustic emission, and scanning electron microscopy (SEM) [18]. However, SEM is an essential tool for the microscopic damage assessment of FRPs during the material design and evaluation process. SEM is an essential tool for fractographic analysis of FRPs, enabling a detailed examination of microstructural damage mechanisms, including fiber breakage, fiber pullout, mixed-mode failure, matrix ductile failure, and matrix brittle failure [19,20]. This information is crucial for understanding the failure behavior of composites and optimizing their design for enhanced performance and reliability [21]. However, the complexity and variability of fractographic features make manual interpretation a time-consuming and labor-intensive process, requiring significant expertise and often leading to subjective assessments. To overcome these challenges, automated, data-driven approaches for microscopic damage assessment have gained attention as they offer a more efficient and consistent alternative [22]. These automated techniques utilize advanced image processing [23] and deep learning algorithms [24] to accurately identify and classify damage features, thereby facilitating a more objective and scalable analysis.

Microstructure analysis of composite materials has seen significant advancements with the integration of deep learning and advanced imaging techniques. For instance, X-ray tomography microscopy combined with deep learning has effectively tracked the microstructure evolution of SiCf/SiC-W-ZrB₂ composites during fabrication, demonstrating accurate reconstruction with errors below 8.31% [25]. Similarly, a deep learning-based method using DeepLabV3+ successfully identified void defects in polymer–matrix composites, linking increased void content to a 27% reduction in interlaminar shear strength [26]. Another approach mapped microstructure images to effective moduli using a convolutional neural network (CNN), providing a novel solution for predicting the mechanical properties of heterogeneous materials [27]. Kopp et al. proposed deep learning-based segmentation of multiclass microscale damage in heterogeneous materials, achieving high agreement with human-segmented data and eliminating nearly 100% of manual segmentation time, accelerating the characterization process by two orders of magnitude [28]. In the void content analysis of the optical microscopy images of composite laminates, a robust CNN-based method outperformed traditional thresholding algorithms and demonstrated improved accuracy across multiple laminate types [29]. High-accuracy segmentation of individual phases in metal matrix composites was achieved by Evsevleev et al. [30] using a deep learning-based U-net architecture, enabling precise extraction of critical microstructural parameters. Automated segmentation of 3D tomography images in fiber-reinforced ceramic composites effectively distinguished phases and matrix cracks, overcoming challenges posed by identical image intensities [31]. An image processing-based approach facilitated rapid, non-destructive characterization of microstructural features in fiber-reinforced composites, quantifying heterogeneity and detecting anomalies without extensive experimental testing [32]. High-resolution back scattered electron (BSE) imaging combined with deep learning provided insights into the microstructural effects of graphene oxide–silica reinforcement in cement composites, demonstrating refined pore structures and altered spatial correlations [33]. Integration of deep learning with focused ion beam–SEM analytics enabled automated, unbiased microstructural analysis of Ni/YSZ cermets, enhancing evaluation accuracy despite poor image quality and common microscopy artifacts [34]. Moreover, a multidisciplinary deep learning approach utilizing U-Net architectures achieved high segmentation accuracy for complex lath-bainite structures in steel, emphasizing the importance of data quality and network visualization for reliable microstructure quantification [35].

Despite significant advancements in the automated analysis of composite microstructures using deep learning, existing methods primarily target individual aspects such as phase segmentation, void detection, or property prediction. A comprehensive approach capable of autonomously assessing multiple damage modes under varying loading conditions for FRPs remains lacking. This study addresses this gap by integrating deep learning models to accurately identify and classify various microscopic damage features in FRPs. In a big data environment, deep learning can significantly enhance material characterization by correlating complex microstructural features and rapidly predicting physical properties [36]. However, training these models requires extensive, high-quality datasets, which are often scarce. To overcome this limitation, the proposed framework employs transfer learning and data augmentation to improve model performance and reliability, offering a scalable solution for holistic damage assessment in FRPs. While the complexity of microstructural features often requires large models for accurate classification, this research focuses on utilizing computationally efficient architectures. Therefore, models such as DenseNet-121, NasNet Mobile, EfficientNet, and MobileNet are employed to achieve precise classification of diverse damage modes with reduced computational demands. This approach provides a robust and scalable solution for material scientists and engineers involved in the design and evaluation of FRPs.

## 2. Proposed Methodology

**Figure 1:**
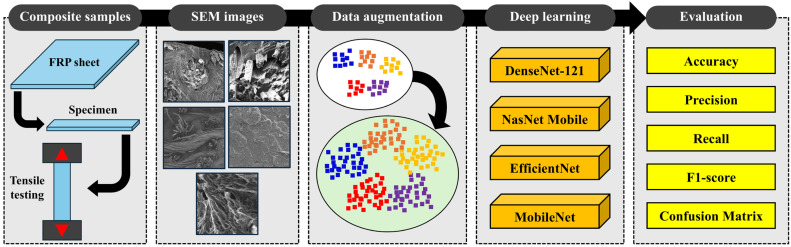
The proposed framework for microscopic damage assessment of FRPs.

This study proposes the use of computationally efficient deep learning models for the microscopic damage assessment of FRPs. Figure 1 shows the proposed methodology that comprises (a) composite sample preparation, (b) collection of SEM images for different failure modes of FRPs, (c) use of data augmentation techniques to increase the size of the dataset, (d) fine-tuning of the pre-trained deep learning modes on the SEM, and (e) evaluation of the proposed models based on multiple evaluation metrics. In the first step, the FRP sheets are prepared and cut into specimen form for tensile testing. The SEM image data are then acquired in the second step from the fractured surfaces of the tensile test specimen, which includes different fiber and matrix-based failure modes of FRPs. The data are then sorted based on each class of failure, and image data augmentation techniques are used to increase the size of the dataset. The data augmentation techniques used for this purpose include vertical flip, horizontal flip, random rotation, zoom-in, random crop, and resize. In the third step, the four computationally efficient deep learning models (DenseNet-121, NasNet Mobile, EfficientNet, and MobileNet) are tuned on the SEM image data. In the final step, the trained models are used to make predictions on unseen data to identify the microscopic failure model of FRPs. The evaluation is performed using accuracy, precision, recall, f1-score, and confusion matrix, which are commonly used for evaluating classification models [37].

### 2.1. Deep Transfer Learning

**Figure 2:**
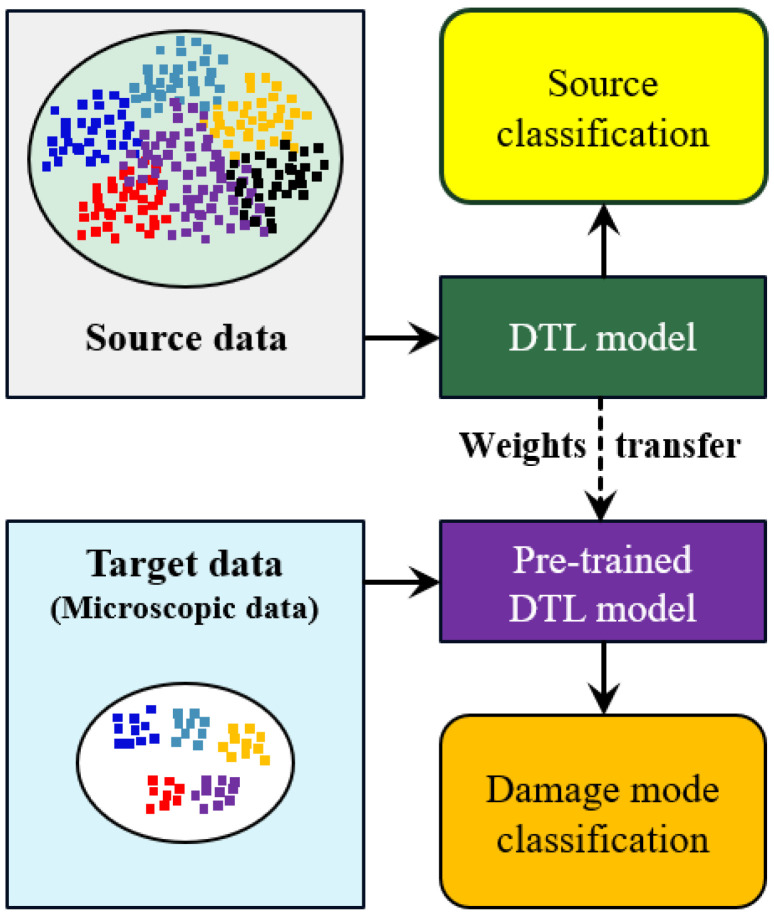
The DTL framework for microscopic damage classification in FRPs, where the DTL model is pre-trained on large source data and then fine-tuned on target SEM data.

Deep learning typically requires a substantial amount of data. Data insufficiency can lead to underfitting during training. Deep transfer learning (DTL) addresses this challenge by pre-training a high-performing model on a large dataset and utilizing its learned features for a specific task with a smaller dataset [38]. The schematic representation of the DTL is shown in Figure 2. The DTL concept has already been implemented for microscopic damage detection of metallic materials. Anidjar et al. [39] utilized ResNet152 and YOLOv5s to classify and identify the cause of failure in titanium Ti-6Al-4V. Similarly, Jones et al. [40] used VGG16-based DTL models to predict fatigue crack growth in SEM images of Ti-6Al-4V. However, the traditional DTL models require high computational resources, with model sizes of more than 200 MB and model parameters of tens of millions [41]. To address the limitations of traditional DTL models, this study proposes the utilization of computationally efficient architectures such as DenseNet-121, NasNet Mobile, EfficientNet, and MobileNet, which have fewer than 10 million parameters and model sizes under 50 MB. These models offer a significant reduction in computational requirements, making them more feasible for real-time microscopic damage assessment and material design optimization. By enabling rapid and accurate analysis of FRP microstructures, these computationally efficient DTL models facilitate the development of advanced FRPs with enhanced mechanical properties and reliability.

#### 2.1.1. DenseNet-121

**Figure 3:**
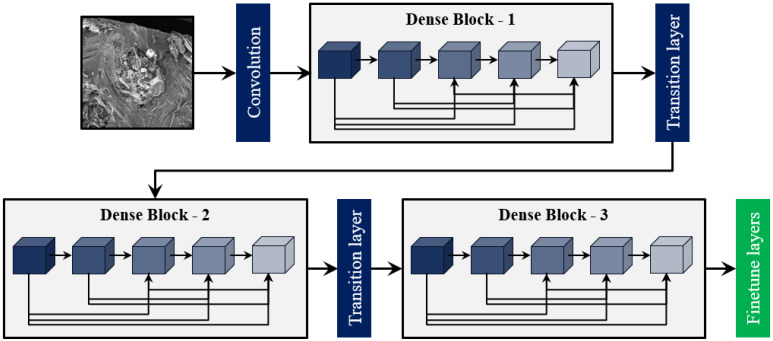
The schematic representation of the DenseNet121-based DTL model.

DenseNet [42] was adopted as the DTL model for microscopic damage assessment of FRPs due to its exceptional performance in ImageNet classification problems [43]. In DenseNet architecture, each layer has direct connections to all subsequent layers, enabling the flow of information and gradients throughout the network. This approach involves the concatenation of feature maps from preceding layers with the feature maps of the current layer, which enhances feature reuse and mitigates the vanishing gradient problem. The architecture is composed of dense blocks, which contain a sequence of layers that include batch normalization, ReLU activation, and convolution operations. Between these dense blocks, transition layers are introduced, consisting of batch normalization, 1 × 1 convolution, and pooling layers, to down-sample the feature maps and control model complexity. A schematic of the DenseNet architecture containing an input layer, a convolutional layer, three dense blocks, two transition layers, and the fine-tuning layer is shown in Figure 3. DenseNet-121 is the most computationally efficient variant of the DenseNet family with 7.89 million parameters; therefore, it has been used in this study for microscopic damage assessment of FRP composites.

#### 2.1.2. NasNet Mobile

**Figure 4:**
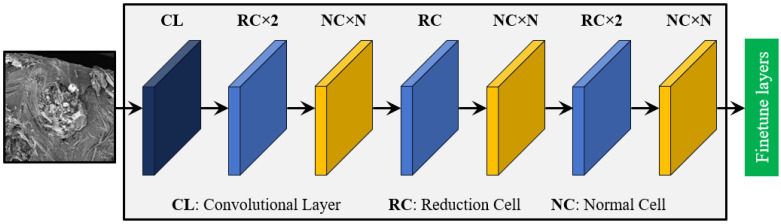
The schematic representation of the NasNet-based DTL model.

NasNet-Mobile (NasNet) is a scalable convolution-based architecture with cells utilizing the reinforcement learning approach proposed by Zoph et al. [44]. Unlike traditional architectures, NasNet is developed using neural architecture search (NAS), which systematically explores the space of possible network structures to identify the most effective configuration. This architecture consists of two fundamental building blocks known as normal cells (NCs) and reduction cells (RCs). These cells are modular sub-networks composed of multiple convolutional layers, designed to be reusable and adaptable. NCs maintain the spatial dimensions of the input, while RCs decrease the spatial resolution, allowing for a flexible and efficient architecture that can be easily tailored to different tasks and computational constraints. NasNet utilizes this modular design by stacking these cells to create a scalable and resource-efficient network suitable for mobile and low-power applications. The schematic representation of the NasNet model is shown in Figure 4, where N is the number of cells optimized on the dataset, and the architectural details of the NCs and RCs can be found in Ref. [45]. The NasNet consists of 5.32 million trainable parameters [46].

#### 2.1.3. EfficientNet

**Figure 5:**
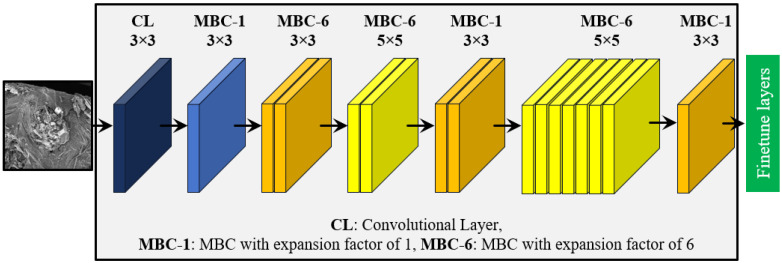
The schematic representation of the EfficientNet-based DTL model.

EfficientNet stands out as the state-of-the-art DTL model, particularly due to its performance on the ImageNet dataset. EfficientNet employs the swish activation function, improving the ability of the model to capture complex patterns and offering better gradient flow and non-linear transformations compared to ReLU [47,48]. EfficientNet aims to build models that are both powerful and computationally efficient by employing compound scaling. This technique uniformly adjusts the network’s depth, width, and resolution, enhancing performance without significantly increasing computational costs. The baseline network is scaled in all dimensions according to a set of predefined scaling factors, ensuring optimal performance under limited resources [47]. The core building block of EfficientNet architecture is the mobile-inverted bottleneck convolution (MBC). The MBC layers function by first expanding the number of channels and then compressing them, effectively creating a bottleneck structure. This reduces the computational cost by limiting the number of channels in the bottleneck compared to the expanded layers. The architecture also utilizes depth-wise separable convolutions, which significantly decrease the number of floating-point operations by a factor of *k*^2^, where *k* represents the kernel size. This combination enables EfficientNet to achieve high performance with fewer computational resources. The EfficientNetB0, being the most compact variant of the EfficientNet family, has been used in this study, which contains 5.40 million parameters [49]. The schematic representation of the EfficientNet model is shown in Figure 5.

#### 2.1.4. MobileNet

**Figure 6:**
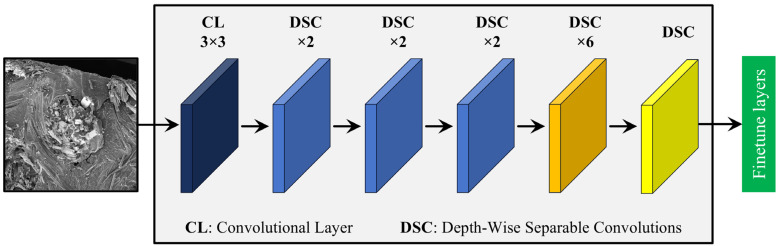
The schematic representation of the MobileNet-based DTL model.

MobileNet is a family of DTL models optimized for efficient use on mobile and embedded devices, characterized by their low computational and memory requirements. The architecture employs depth-wise separable convolutions (DSCs) to reduce calculations and the number of parameters to perform classification or image recognition tasks [50]. DSC decomposes a standard convolution into two operations: a depth-wise convolution, which filters each input channel independently, and a pointwise convolution, which combines these filtered outputs using 1 × 1 convolutions. This separation significantly reduces the number of parameters and computational cost, making the network more efficient. MobileNet further optimizes computational efficiency with bottleneck layers that compress the input feature maps before processing them through the convolutional layers. The MobileNet family, including MobileNet, MobileNetV2, and MobileNetV3, provides additional architectural improvements such as inverted residuals and linear bottlenecks to enhance performance across various applications while maintaining low computational requirements [51,52]. The basic MobileNet model shown in Figure 6 with 4.2 million parameters has been chosen for this study due to its balance between accuracy and computational efficiency, making it suitable for deployment in resource-constrained environments while still providing reliable performance for image classification.

## 3. SEM Dataset and Data Pre-Processing

### 3.1. SEM Dataset Description

**Figure 7:**
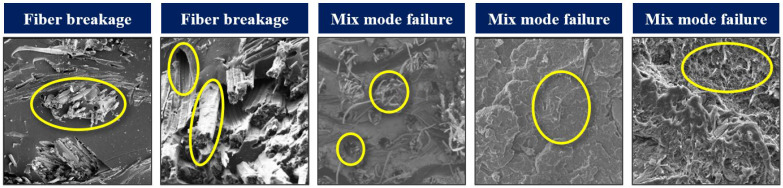
The representative SEM images showing different failure modes in FRP composites used in this study; the circled areas show the specific region of the damage mode.

The SEM data utilized in this study focus on surface-level defects rather than internal damage. The SEM dataset comprises microscopic images collected from fractured tensile test specimens of FRPs, offering insights into surface microstructural damage mechanisms. However, it is important to note that internal damage may result from the coupling of various damage mechanisms, which cannot be fully captured by SEM alone. Therefore, this study is focused only on the SEM image-based damage assessment to provide detailed insights into surface-level defects. The dataset is collected from the published literature, which includes images of FRPs with both synthetic and natural fibers, as well as composites with synthetic and bio-based resins [20,53,54,55]. The FRPs in the selected literature were fabricated using manufacturing techniques such as vacuum-assisted resin transfer molding (VARTM) and hot press compression molding. This diverse data composition captures a broad range of microstructural damage mechanisms, providing a comprehensive representation of damages that can occur in FRPs. The collected dataset is classified into five distinct damage modes, comprising three fiber-based and two matrix-based models. These damage modes are shown and highlighted in Figure 7, while a brief description of each mode is as follows:

1. **Fiber Breakage (FB)**: This mode occurs when fibers fracture due to high tensile stress, indicating a critical failure in the load-bearing capacity of the composite. The fracture surfaces in this mode exhibit characteristic brittle fracture patterns perpendicular to the fiber axis.
2. **Fiber Pullout (FP)**: In this mode, fibers are disengaged from the matrix without fracturing, suggesting inadequate fiber–matrix interfacial bonding. This failure mechanism is indicative of suboptimal stress transfer between matrix and fibers, which compromises the overall mechanical integrity.
3. **Mixed-Mode Failure (MMF)**: This complex failure involves a combination of fiber breakage and pullout. It reflects a scenario where simultaneous interfacial debonding and fiber fracture occur.
4. **Matrix Brittle Failure (MBF)**: This damage mode exhibits minimal plastic deformation and generates sharp, brittle fracture surfaces that propagate rapidly through the matrix. Stress concentrations or abrupt loading conditions typically initiate this type of failure, leading to catastrophic fracture due to the material’s low fracture toughness. MBF is often associated with the use of a thermoset matrix in FRPs.
5. **Matrix Ductile Failure (MDF)**: This damage mode exhibits significant plastic deformation before fracture, indicating that the matrix material undergoes extensive yielding before failure. It is commonly associated with a gradual failure process and is typically observed in polymer matrices that possess high ductility. MDF is often associated with the use of a thermoplastic matrix in FRPs.

The inclusion of both synthetic and natural fibers, along with synthetic and bio-based resins, provides a diverse and representative dataset. This diversity is beneficial for developing more generalized and robust DTL models capable of accurately identifying a wide range of damage modes across different FRPs. Such generalized models are crucial for enhancing the reliability and scalability of automated microscopic damage assessment in FRP composites, thereby facilitating a more efficient material design process.

### 3.2. Data Augmentation

Due to the limited availability of SEM images in the literature, data augmentation is a critical strategy to expand the dataset and enhance the performance and robustness of DTL models. Data augmentation not only addresses the issue of limited data but also helps improve the generalization capability of the model by simulating variations in damage patterns and conditions that may not be exhibited by the original dataset. The following five data augmentation techniques have been applied to expand and diversify the training dataset: vertical flip, horizontal flip, random rotation, zoom in, and random crop and resize. The vertical flip technique flips the images along the vertical axis, creating mirror images of the original data. It is particularly useful in simulating different orientations of damage features, thereby helping the model to generalize across vertically varied fracture surfaces. The horizontal flip introduces horizontal variations in the dataset. This is crucial for training the model to recognize damage patterns that may appear differently depending on the direction of loading or observation, which is essential for accurate classification of damage modes under various loading conditions. The random rotation augmentation rotates the images by arbitrary angles, enhancing the model’s ability to recognize damaged features from different perspectives. This technique is vital for scenarios where fracture surfaces may be viewed at varying angles, ensuring that the model can accurately identify and classify damage regardless of the sample’s orientation during SEM imaging. The zoom-in technique magnifies specific regions of the image, emphasizing fine details of the damage patterns. This augmentation is particularly beneficial for microscopic damage assessment as it allows the model to focus on intricate microstructural features of both fiber and matrix damage modes. The random crop and resize augmentation randomly crops a region from the original SEM image and resizes it to the original dimensions, introducing variations in scale and perspective. It effectively simulates different magnifications and viewpoints, enabling the model to robustly identify damage features that may appear at varying scales due to changes in SEM magnification settings. A total of 1110 SEM images were obtained after data augmentation, which were then resized to a pixel size of 224 × 224, which is the input size for all the computationally efficient DTL models proposed in this study. The dataset was then split into 80% for training and 20% for testing. Within the 80% training data, a 5-fold cross-validation strategy was employed, where the data were further divided into five sets. Among the five training sets, four were used as training sets and one as a validation set during each fold. This approach ensured that the model was exposed to all data during training while also minimizing the risk of overfitting to any particular subset of the data. The training results for the 5-fold cross-validation are presented in Section 4, while the test data are used as unseen data to validate the proposed model in Section 5.

## 4. Model Training Results

The computationally efficient DTL models presented in Section 2.1 were trained using the prepared dataset. Moreover, to maintain consistency among the proposed pre-trained models, the layer weights for all models were frozen, and the three additional layers were added to adapt the models for microscopic damage assessment. The additional layers include a global average pooling (GAP) layer, a dense layer, and a classification layer. The GAP layer averages the spatial dimensions of the feature maps extracted from DTL models, effectively reducing the data dimensionality while retaining the essential information. This operation helps in minimizing overfitting and enables a smoother transition from feature extraction to classification. Following the GAP layer, a fully connected dense layer with 1028 neurons is added, which processes the compact feature representation into a dense vector, enabling the model to learn complex patterns and relationships specific to the different microscopic damage modes in FRP composites. Various configurations with different neuron counts were tested, but the dense layer with 1028 neurons consistently yielded the best performance. Therefore, it has been selected for the final model configuration of DTL models in this study. Finally, the final classification layer is added with five neurons using a SoftMax activation to output the probability distribution across the five predefined damage modes. The classification layer converts the output of the dense layer into specific damage modes, allowing for accurate and precise classification of microscopic damage modes within the SEM dataset.

All four DTL models (DenseNet121, NasNet, EfficientNet, and MobileNet) were trained using a consistent set of hyperparameters to ensure a standardized evaluation of their performance for microscopic damage assessment of FRPs. Each model was trained for 40 epochs with a batch size of 32, which provides a sufficient number of iterations for effective learning while mitigating the risk of overfitting. The Adam optimizer, with a learning rate of 0.001, was employed due to its adaptive learning capabilities, which allow for efficient convergence during training. The loss function utilized was sparse categorical cross-entropy, suitable for multi-class classification problems where the objective was to accurately classify the different microscopic damage modes. To ensure robust model evaluation and reduce bias, a 5-fold stratified cross-validation approach was implemented, maintaining class balance across training and validation sets. This rigorous training procedure was designed to check the generalization ability of the DTL models.

**Figure 8:**
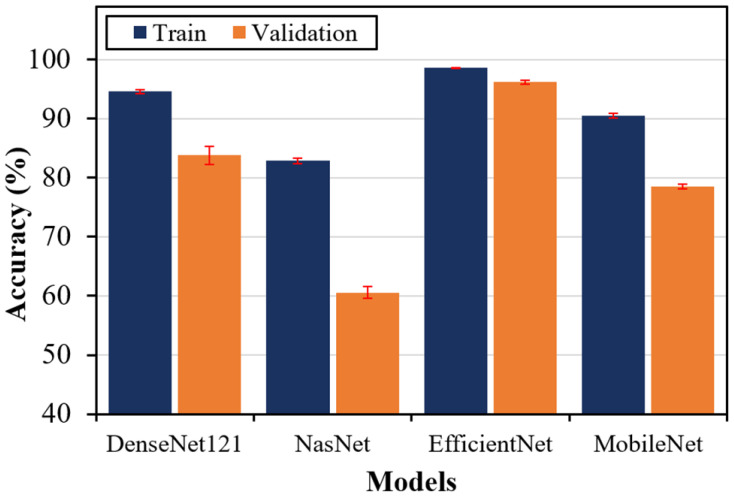
The training and validation accuracy results for the DTL models based on stratified 5-fold validation.

**Figure 9:**
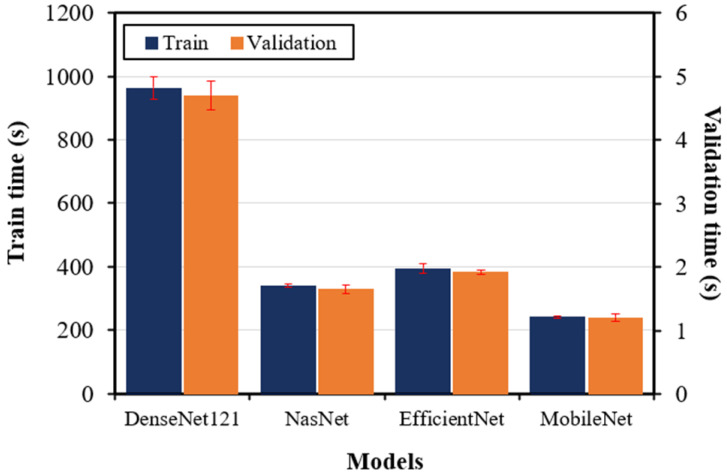
The training and validation times for the DTL models based on stratified 5-fold validation.

Figure 8 and Figure 9 present a comprehensive comparison of the training and validation results in terms of accuracy and computational time, respectively, of the four DTL models. Figure 8 shows that EfficientNet achieved the highest training and validation accuracies of 98.71% and 96.23%, respectively. This close alignment between training and validation accuracies indicates the robustness of the EfficientNet model. The DenseNet121 model also demonstrated a good training accuracy of 94.61%; however, the low validation accuracy of 83.80% indicates overfitting. The overfitting is further prominent in the MobileNet and NasNet models. The increased overfitting in these models is potentially due to their reduced model complexity. Figure 9 provides a comparative analysis of the computational resources of each DTL model in terms of training and validation time. DenseNet121, despite achieving good accuracy, exhibited the longest training duration, indicating substantial computational resource requirements, which may limit its practical applicability in real-time microscopic damage assessment scenarios. NasNet demonstrated significantly lower training time but at the expense of accuracy, showing its limitations in capturing complex damage features present in SEM images. EfficientNet demonstrated the most balanced metrics, providing high classification accuracy with a moderate computational time. MobileNet showed the lowest training and validation times but exhibited a noticeable trade-off in predicting damage modes, making it unsuitable for applications demanding high accuracy of complex microstructural damage modes in FRP composites. These findings emphasize the use of the EfficientNet model for achieving an optimal balance between computational efficiency and accuracy in predicting complex microstructural damage modes in FRP composites.

## 5. Model Validation and Discussion

**Figure 10:**
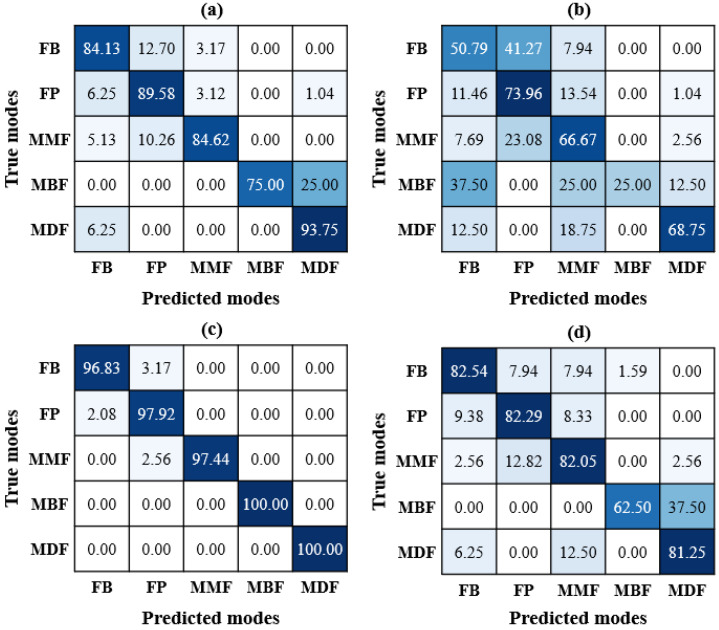
CM using unseen test data: (**a**) DenseNet121, (**b**) NasNet, (**c**) EfficientNet, and (**d**) MobileNet models.

This section provides validation and discussion on the proposed DTL models based on the unseen test dataset using multiple evaluation metrics such as precision (P), recall (R), f1-score (F1), and confusion matrix (CM). A detailed description of these evaluation metrics can be found in Ref. [2]. The evaluation of models on unseen data is useful in avoiding overfitting the training data and determining the generalization ability of the model on new data. The proposed DTL models showed testing accuracies of 86.94%, 63.96%, 97.75%, and 81.53% for DenseNet121, NasNet, EfficientNet, and MobileNet, respectively. These results again demonstrate that the EfficientNet model is well-suited for microscopic damage assessment of FRPs, as it can effectively capture the damage-sensitive patterns from SEM images. Further evaluation of the microscopic damage assessment of FRP composites is made using the CMs shown in Figure 10. The CMs present the classification performance of each DTL model, where the diagonal cells represent correct classifications (true positives for damage modes) and off-diagonal cells indicate misclassifications (false positives and false negatives). In Figure 10a, DenseNet121 shows good accuracy in classifying most damage modes, with minor confusion between FB and FP, suggesting some overlap in feature representation. The DenseNet model also confuses the brittle failure of the matrix with ductile failure by 25%. Figure 10b for NasNet shows lower diagonal values, particularly for FB and MBF. This indicates reduced classification efficiency and higher confusion across damage modes, likely due to the limited feature extraction capabilities of NasNet for complex microstructural patterns in FRPs. Figure 10c demonstrates that the EfficientNet model exhibits excellent performance, achieving near-perfect accuracy for all fiber-based damage modes and 100% classification for matrix-based failures (MBF and MDF). This demonstrates the robust feature extraction ability of EfficientNet by effectively differentiating between sensitive variations in damage modes. Figure 10d shows that the MobileNet model can predict the fiber-based damage modes with around 82% accuracy but struggles to differentiate the matrix-based failure modes. Overall, the EfficientNet model demonstrated superior and more consistent performance in accurately identifying both fiber- and matrix-based microscopic damage modes, highlighting its potential for automated damage assessment in FRP composites. Therefore, due to the effective performance of the proposed DTL models, the developed models can be integrated into the material design process, allowing material scientists to autonomously predict the five damage modes in FRPs. This capability would enable more informed material selection and fabrication methods, ultimately enhancing the design, durability, and performance of composite structures. However, the application of the proposed approach is restricted to the material designing phase due to the use of surface-level defects only. Therefore, it cannot be used directly for in-service FRP structures. Nevertheless, if the training of these DTL models is extended to practical scenarios, it is expected that they could also be applied for in-service damage monitoring and assessment, providing a more comprehensive evaluation of FRP structures throughout their lifecycle.

To further strengthen the comprehensive evaluation of DTL performance, additional assessment metrics such as P, R, and F1 were evaluated for each microscopic damage mode. Table 1 showcases these metrics for DenseNet121, NasNet, EfficientNet, and MobileNet models. The EfficientNet model consistently outperformed other models across all damage modes, achieving the highest *p* values for FB, FP, and MBF with scores of 96.83%, 96.91%, and 100.00%, respectively. Similarly, both brittle and ductile matrix failure were also identified with 100.00% P, R, and F1 values. This indicates a high proportion of correctly identified positive cases relative to all predicted positives, highlighting the superior ability of the EfficientNet model to accurately classify the microscopic damage modes. DenseNet121 performed consistently well across most damage modes, especially MBF. However, the overall values for P, R, and F1 were relatively lower compared to the EfficientNet model. NasNet demonstrated the lowest performance, with P, R, and F1 values falling mostly below 70%. This suggests that the reduced model complexity of NasNet hindered its ability to capture complex microstructural features from SEM images. MobileNet achieved moderate performance with P, R, and F1 values inconsistently fluctuating between 68.09 and 88.76%. This suggests that the MobileNet model is unable to generalize on the unseen test data. Overall, these results signify that the pre-trained EfficientNet model delivers superior performance in microscopic damage assessment of FRP composites compared to DenseNet121, NasNet, and MobileNet, making it the most effective model for accurately detecting and classifying complex damage features in SEM images with limited computational resources.

## 6. Conclusions

This study proposed a DTL-based framework to classify microscopic damage modes in FRP composites using SEM images. This investigation compared the performance of four pre-trained DTL models, namely DenseNet121, NasNet, EfficientNet, and MobileNet, with EfficientNet achieving the highest accuracy of 97.75% on the unseen test dataset. DenseNet121 and MobileNet followed with accuracies of 86.94% and 81.53%, respectively, while NasNet performed the lowest at 63.96%. By accurately identifying damage modes such as FB, FP, MMF, MBF, and MDF, the models demonstrated their capability to automate the damage classification process effectively. Furthermore, the EfficientNet model showed 100% accuracy in differentiating the brittle and ductile failure modes present in the matrix material of FRPs, showing its suitability for material design applications. Therefore, the EfficientNet model offers a practical tool for material scientists, allowing them to assess damage autonomously during the material design phase, which can aid in the optimization of material selection and fabrication processes. The integration of EfficientNet in the material design would reduce the labor-intensive nature of manual assessments of SEM images and enhance the consistency of damage classification. Thus, the autonomous evaluation of the SEM images could contribute to improving the durability and performance of FRP composites in critical industries, such as aerospace and automotive industries. However, a key limitation of this work is that it focuses exclusively on surface-level damage, as SEM primarily captures surface defects. To extend the applicability of the proposed approach, integrating other non-destructive evaluation techniques, such as μCT or acoustic emission, could enable the detection of internal damage in FRPs, providing a more comprehensive damage assessment framework. Moreover, this study focused on five failure modes in FRPs; future work will incorporate other critical damage scenarios such as delamination, fiber–matrix debonding, and fiber bridging. Expanding the model to include these will create a more comprehensive framework for autonomous damage assessment, enhancing the applicability of the approach across a wider range of damage scenarios in FRP composites.

## Figures and Tables

**Table 1:** The performance comparison of the DTL models for damage assessment of FRP composites using P, R, and F1 metrics.

Damage Model	DTL Model	P (%)	R (%)	F1 (%)
**FB**	DenseNet121	85.48	84.13	84.80
	NasNet	62.75	50.79	56.14
	EfficientNet	96.83	96.83	96.83
	MobileNet	82.54	82.54	82.54
**FP**	DenseNet121	87.76	89.58	88.66
	NasNet	66.98	73.96	70.30
	EfficientNet	96.91	97.92	97.41
	MobileNet	88.76	82.29	85.41
**MMF**	DenseNet121	86.84	84.62	85.71
	NasNet	53.06	66.67	59.09
	EfficientNet	100.00	97.44	98.70
	MobileNet	68.09	82.05	74.42
**MBF**	DenseNet121	100.00	75.00	85.71
	NasNet	100.00	25.00	40.00
	EfficientNet	100.00	100.00	100.00
	MobileNet	83.33	62.50	71.43
**MDF**	DenseNet121	83.33	93.75	88.24
	NasNet	78.57	68.75	73.33
	EfficientNet	100.00	100.00	100.00
	MobileNet	76.47	81.25	78.79

## Data Availability

The data will be made available upon request.
